# Study of house-level risk factors associated in the transmission of Indian Kala-azar

**DOI:** 10.1186/1756-3305-3-94

**Published:** 2010-10-12

**Authors:** Shreekant Kesari, Gouri Sankar Bhunia, Vijay Kumar, Algarswamy Jeyaram, Alok Ranjan, Pradeep Das

**Affiliations:** 1 Department of Vector Biology and Control, Rajendra Memorial Research Institute of Medical Sciences (ICMR), Agamkuan, Patna-800 007, Bihar, India; 2 Regional Remote Sensing Service Centre (ISRO), IIT Campus, Kharagpur, West Bengal

## Abstract

**Background:**

In visceral leishmaniasis (VL), phlebotomine vectors are the main target to reduce for control measures. An attempt has been taken to delineate the association between *Phlebotomous argentipes *and housing characteristics between two districts e.g. endemic and non-endemic.

**Methods:**

A cross-sectional survey was conducted on 240 households for both the endemic (Vaishali district) and non-endemic (Lohardaga district) site. Logistic regression analysis was used to identify factors related to housing characteristics influencing suitable habitats for *P. argentipes*. Vector density estimated using a CDC light trap.

**Results:**

The proportion of *P. argentipes *in both endemic and non-endemic areas was significantly much higher (P < 0.001) when compared with the proportion of *Sergentomiya *and *P. papatasi*. The results of multilevel logistic regression analysis showed that mud plastered wall (*P value = 0.001)*, mixed dwelling (*P value = 0.002*) and area (*P value = 0.001*) were strongly associated with the presence of vectors.

**Conclusion:**

Result of the studied household characteristics provides an accurate, rapid assessment of house-level variation in risk. The results also have implications for maximizing surveillance efficacy of sandflies, which is likely to become increasingly important while formulating any control strategy.

## Background

Visceral leishmaniasis (VL) or Kala-azar is a serious public health problem and has worldwide distribution. It is caused by the intracellular protozoan parasite of genus *Leishmania*, which is transmitted by phlebotomine sandflies. A large portion of 500, 000 annual cases and 60,000 deaths occur in the poor rural communities within the Indian subcontinent [1,2]. At present, in India 31 out of 38 districts in Bihar, 10 out of 24 districts in West Bengal and 4 districts bordering Bihar (in Uttar Pradesh) are endemic at different levels. High incidence of VL in India is reported during the monsoon & post monsoon season [3]; these coincide with vector presence and a high proportion of the parous vector. It indicates an increase man-vector contact during warmer part of the year also [4].

*Phlebotomous argentipes*, the vector of kala-azar in the Indian subcontinent [5], being insects of warm climates are generally abundant during monsoonal part of the year [6]. The vector, living inside the houses (endophilic species), causes a large burden of disease [7,8]. Large scale control programmes, mainly based on household spraying of residual insecticides, have achieved cost-effective local eradication of domestic vectors and interruption of Kala-azar transmission in different parts of the world [7-12], but recent reports, Chowdhury et al., [13] suggests that in India desired impact of Indoor Residual Spray (IRS) is not being achieved due to several reasons.

Previously, very little work has been done at household level and they have mentioned poor housing construction as characteristics of the study area where visceral leishmaniasis was prevalent and vector density is also high [14-16]. In addition, a previous study has assessed risk factors based on low socio-economic group of endemic areas that are vulnerable to disease; they have mentioned nothing about the construction and physical characteristics of the houses [17]. These analyses have usually been carried out as well-controlled localized studies in a Kala-azar endemic area of Bihar. This study provides an opportunity to measure the association between the characteristics of individual houses over the endemic and non-endemic region of Kala-azar, and risk of infestation with *P. argentipes*. This study can serve to identify of house-level risk factors which may provide additional information on risk that is relatively rapid and inexpensive to collect, and complements other sources of risk information that are more specific; and this will ultimately help in the successful implementation a control strategy.

The present study evaluates standardize cross-sectional survey of vector infestation and physical characteristics of the houses within the Kala-azar endemic and non-endemic region in India. It should be therefore support current efforts to scale-up control activities within the region.

## Materials and methods

### Study area

Two study sites were selected: Vaishali district (Latitude 25°43'N -26°00'N to longitude 85°04'E -85°38'E), one of the high endemic areas of Kala-azar in north Bihar, as endemic foci and Lohardagga district (23°30'N -23°40'N latitude to 84°40'E -84°50'E longitude), having no cases during the last decade, as non-endemic foci. The total areas of the selected village in endemic and non-endemic sites are 7008.74 and 15829.01 hectares respectively. The numbers of households are approximately 8206 and 4221 having populations of 54321 and 23082, in the endemic and non-endemic site, respectively.

### Demographic survey

A cross-sectional descriptive study was designed and conducted in the two districts from October 2007 to July 2008 in an endemic site (Vaishali District) and February 2009 to July 2009 in a non-endemic site (Lohardaga District).

In each district, twenty four villages were selected randomly to carry out the research work. In each village 10 houses were selected randomly and were sampled for indoor day-resting sandflies by a Communicable Disease Centre (CDC) Light trap. Thus two hundred and forty households of the twenty four villages were taken from Vaishali district, suspected to have VL cases and two hundred and forty households of the twenty four villages were taken from Loharadaga district with no recorded VL cases.

### Sandfly Collection

Sandflies were collected through Communicable Disease Centre (CDC) light trap from the living rooms as well as cattle sheds respectively from the endemic and non-endemic houdeholds. Collected sand flies were preserved in 70% alcohol and transported to the laboratory; sandfly number, type and gender, and vector species were also recorded for each site in the laboratory.

### Statistical analysis

Since multilevel cluster sampling design was used taking a village as primary selection unit (PSUs) and randomly selected houses from each cluster, Stata version 10.0 'xt' command was used for performing multilevel logistic regression analysis to investigate the effect of the house-level characteristics on the presence or absence of vectors in the household. The majority of housing characteristics are categorical variables (e.g. such as house construction (types of Floor, Wall, and Roof), presence of cattle shed, and cattle population) (Table 1). In this study, all the housing characteristics recorded in the survey was considered as independent variables, whereas, presence or absence of vector was considered as dependent variable. Before applying the logistic regression analysis, univariate analysis was performed for each independent variable with the presence/absence of vector (Table 2). Variables, which were found significant in the course of univariate analysis with p-values less than equal to 15%, were retained for the logistic regression analysis for cluster design. In order to check the interaction effect, the interaction terms of all the possible combination of significant variables as observed in univariate analysis were put simultaneously in the model, and insignificant terms were removed stepwise from the model in order to get the final model. The result of final model with intra-class correlation (rho) is presented in Table 3.

**Table 1:** House-level data considered for the presence or absence of *P. argentipes *in the study site (Both endemic and non-endemic).

House-level characteristics	Description	Units
Sandfly Presence/Absence	Sandfly collected through CDC light trap	Counts (In each Villages per 10 house)
House Floor	**Floor -**	
	1. Cemented	**0**= Cemented (Ref.)
	2. Mud	**1**= Mud
Wall	**Wall-**	
	1. Brick with cemented and Plastered	**0**= Brick with cemented and plastered (Ref.)
	2. Brick	**1 = **Brick
	3. Thatched	**2 **= Thatched
	4. Mud and Brick with Mud	**3**= Mud and Brick with Mud
Roof	**Roof-**	
	1. Cemented and Asbestos	**0**= Cemented and Asbestos (Ref.)
	2. Tiles and Cuprile	**1**= Tiles and Cuprile
	3. Thatched	**2**= Thatched
Presence of cattleshed	**Cattleshed-**	
	1. No cattle Population	**0**= No cattle Population (Ref.)
	2. Separate	**1 = **Separate
	3. Mixed Dwelling	**2 = **Mixed Dwelling
Cattle Population	**Cattle population-**	
	1. No cattle Population	**0 = **No cattle population/Goat (Ref.)
	2. Presence of Cattle population	**1 = **Cow/Buffalo
Area	**Area**	
	1. Non-endemic	**0 = **Non-endemic (Ref.)
	2. Endemic	**1**= Endemic

(Ref.) = Reference category

**Table 2:** Association of household variables with the presence of sandfly (Univariate analysis)

Characteristics	Crude odds ratio (95% CI)	*P*-*value*
House floor (mud)	1.39 (0.67-2.87)	0.367
House wall (mud)	1.44 (1.13-1.83)	0.004
House roof (thatched)	1.60 (1.14-2.25)	0.008
Presence of cattleshed (yes)	1.49 (0.72-3.08)	0.274
Location of Cattleshed (mixed dwelling)	1.68 (1.11-2.53)	0.015
Area (endemic)	1.38 (0.92-2.05)	0.113

**Table 3:** Logistic regression analysis of household variables with the presence of sandfly

Variables	Adjusted odds ratio (95% CI)	*P-value*
House wall	1.71 (1.33-2.20)	0.001
Location of Cattleshed	1.60 (1.18-2.14)	0.002
Area	2.22 (1.37-3.59)	0.001
Intraclass Correlation	0.015 (0.0013-0.625)	0.326

## Results

### Sandfly capture and its distribution

During October 2007 to July 2009, a total of 535 sandflies were collected from the twenty four villages (240 households) in an endemic site (Valshali District), which consisted of 53.90% males and 46.10% females. Similarly, from the non-endemic sites (Lohardaga District), a total of 268 Phlebotomine sandflies were collected from twenty four villages (240 households), among them 57.41% were male and 42.59% were female. The major percentage of phlebotomine sandflies collected from endemic and non-endemic sites belong to *P.argentipes *(in an endemic site -65.98% and non-endemic site-61.48%), followed by *Sergentomiya *(32.53% in endemic site and 38.52% in non-endemic site) and very small percentage of *P. papatasi *(1.49% in an endemic site only). In an endemic area, out of ten houses in each village, 60% of houses had shown the presence of vectors, whereas, in non-endemic area only 30% of houses had the vector present.

Overall, the proportion of *P. argentipes *in both endemic and non-endemic areas was significantly much higher (P < 0.001) as compared with the proportion of the *Sergentomiya *and *P. papatasi*. However, there was no significant difference in the proportion of *P. argentipes *and *Sergentomiya *in the endemic area as compared with the non-endemic area. This indicates that the distinction of different species of sandfly is similar in endemic and non-endemic area.

### Risk factors by house-level characteristics

First univariate analysis was performed for estimating crude odds ratios with 95% confidence intervals (Table 2). Among all the variables, house floor (p = 0.367) and presence of cattle shed (0.274) were not found significantly associated with presence of sandfly. Significantly associated variables with p-values less than or equal to 0.15 were retained for logistic regression analysis putting all the variables with the possible combination of interaction terms into the model and then removing the least significant variables for the final model. The result is presented in Table 3 with adjusted odds ratios with 95% confidence intervals.

The results (Table 2) showed that mud plastered wall (*P value = 0.004)*, thatched roof (*P value = 0.008*), mixed dwelling (*P value = 0.015*) and area (*P value = 0.113*) were found associated with presence of absence of vectors.

The final model (Table 3) indicated that the type of house wall, especially mud plastered wall (OR-1.71; *P-value = *0.001), mixed dwellings i.e. cattle and human being (OR-1.60; *P-value = *0.002) living together in high endemic area (OR-2.22; *P-*value = 0.001) of Kala-azar could be the possible independent risk factors for presence of vector population without having any significant joint effect. The intra-class correlation (ρ = 0.015, 95% CI, 0.0016-0.625) was very low indicating heterogeneity among the PSUs (villages) but not statistically significant (p = 0.318).

## Discussion

The cross-sectional survey of *P. argentipes *infestation in India provides an important source of information to inform control programmes for Kala-azar, and specifically to identify house-level risk factors for *P. argentipes*. The most important characteristics of the survey are (i) standardize collection method of *P. argentipes *and housing quality, (ii) geographic coverage of Kala-azar endemic and non-endemic region, and (iii) use of non-endemic region for positive control services, in a manner that could be replicated in future large scale surveys and monitoring programmes.

Analysis of the survey results indicates statistically significant relationships between specific house-level characteristics and the domestic infestation with the phlebotomine vector. The results of our study, including housing structure with mud plastered wall, thatched roof, mixed dwelling and area are suitable for breeding and the propagation of *P.argentipes*. Hence, they may be considered as a risk factor for disease transmission. Generally, poor housing construction was found to be characteristics of the study area where visceral leishmaniasis was prevalent and vector density is also high. The reports of earlier workers, also support these findings and have been reported that mud plastered household and cattle shed are suitable for the development of phlebotomine species in Indian sub-continent [16,17], Brazil[18] and Kenya [19] respectively. The largest odds ratios, however, are associated with wall types of the houses. Hence, improving housing construction and hygienic condition of houses, consisting of concrete floor, cement plaster and brick walls, and cemented or asbestos roof might be predominantly to reduce the vector density. These obviously have implications in terms of raising overall living conditions which could decrease the transmission and vector presence and/or absence.

Favorable transmission conditions resulting in significant disease may thus be associated with the presence of cattle sheds [20]. Our study has confirmed that cattlesheds, explicitly, mixed dwelling/interconnection of the houses (*P value *= 0.002) strongly associated with the presence/absence of vector. Sandflies were collected from the cattle shed of endemic and non-endemic sites, and in most instances the cattle sheds were poorly ventilated with high organic contents (cow dung and urine of the cattle) and loose wooden planks. Thus it appears that a cattle shed provides a surrogate effect for breeding and resting site of *P.argentipes*, and to ensure continuous transmission of infection to humans. Hence, the cattle population could also influence the presence of vector and transmission of the disease and thus increase exposure to infection as has been previously observed in Bihar [21,22], in Nepal [23] and in Bangladesh [24]. However, the result of logistic regression analysis in our study showed a significant relationship with the location of cattle shed and sandfly presence.

The results of this study have implications for maximizing surveillance efficacy of sandflies, which is likely to become increasingly important in formulating any control strategy. In order to maximize efficacy, however, it is also essential to investigate risk patterns over large spatial scale, and to combine vector data with other sources of risk information. These analyses suggests that future surveys could capture a large proportion of the inter-house variability in disease risk by recording a limited number of key structural factors, and that most important of these (wall, roof, and cattle shed type) could be recorded without needing to enter individual houses. Further analysis will investigate environmental determinants of the geographic distribution of vectors, as well as the inter-relationship between measures of vector infestation and infection risk in humans, in order to optimize allocation of surveillance and control effort. It is the first experimental proof regarding risk factors associated with hose-level characteristics involved in the propagation and multiplication of Kala-azar vector in India.

## Competing interests

The authors declare that they have no competing interests.

## Authors' contributions

SK carried out in the article's conceptualization and conducted the literature review and the design of this study. GSB participated in field data collection, analyzed, interpreted the compiled data and drafted the manuscript. VK helped to draft the manuscript. AR participated in statistical analysis. AJ helped to interpret the results and co-ordination in its design. PD helped envisage this research and contributed to the analysis and interpretation of the results. All authors read and approved the final manuscript.
